# Diagnosis and Endovascular Management of Transplant Renal Artery Stenosis: A Retrospective Two-Decade Study

**DOI:** 10.7759/cureus.80393

**Published:** 2025-03-11

**Authors:** Brankica Spasojevic Dimitrijeva, Milan Djukic, Ivana Gojkovic, Srdjan S Nikolovski, Dragan Sagic, Polina Pavicevic, Tijana Radovic, Dragan Vasic, Oliver Radmili, Igor Stefanovic, Mirjana Kostic, Mirjana Cvetkovic

**Affiliations:** 1 Nephrology, University Children’s Hospital, Belgrade, SRB; 2 Faculty of Medicine, University of Belgrade, Belgrade, SRB; 3 Cardiology, University Children’s Hospital, Belgrade, SRB; 4 Pathology and Laboratory Medicine, Cardiovascular Research Institute, Loyola University Chicago, Chicago, USA; 5 Invasive Diagnostics and Therapy, Institute for Cardiovascular Diseases “Dedinje”, Belgrade, SRB; 6 Radiology, University Children’s Hospital, Belgrade, SRB; 7 Clinic for Vascular and Endovascular Surgery, University Clinical Centre of Serbia, Belgrade, SRB

**Keywords:** angioplasty, blood pressure, glomerular filtration rate, kidney transplantation, renal artery obstruction, stents

## Abstract

Introduction

Transplant renal artery stenosis (TRAS) is a potentially treatable posttransplant complication, primarily presenting with arterial hypertension and allograft dysfunction. Its prevalence in children with posttransplant hypertension ranges from 5% to 15%. Diagnosis is typically made through invasive angiography following suspicion raised by echo Doppler findings. Treatment options include medical therapy, percutaneous transluminal angioplasty (PTA)/stenting, and surgical revascularization. This study aimed to assess the efficacy, complications, and outcomes of PTA/stenting procedures in children with TRAS.

Methods

We reviewed all pediatric patients who underwent renal transplantation in Serbia between June 2001 and February 2023 to identify cases of TRAS treated with PTA. Statistical analysis was performed to compare pre- and post-intervention arterial vessel diameters, serum creatinine levels, estimated glomerular filtration rate (eGFR), mean blood pressure, systolic and diastolic blood pressure indices, and the number of antihypertensive medications used.

Results

Seven patients underwent PTA with or without stent placement for TRAS. None were treated solely with medical therapy or surgical intervention. The overall prevalence of TRAS was 6.32%, higher in cadaveric transplants (11.11%) compared to living-related transplants (3.39%). Of the seven patients, five underwent PTA alone, while two required stent placement. Two of the five PTA patients required re-interventions, resulting in a total of seven angioplasty procedures. No complications occurred following the procedures. After a mean follow-up of 56.86 ± 45.76 months, patients demonstrated improved blood pressure control and reduced use of antihypertensive medications. While the mean eGFR showed a nonsignificant improvement, one patient with severe concomitant cytomegalovirus disease progressed to grade IV chronic kidney disease.

Conclusions

PTA, with or without stenting, appears to be an effective and safe treatment for TRAS in children, with immediate and intermediate-term results comparable to those reported in the literature. Stent placement may be particularly suitable for adolescents who have completed their growth phase.

## Introduction

Transplant renal artery stenosis (TRAS) can lead to posttransplant arterial hypertension, as well as allograft dysfunction and premature loss. Its reported prevalence in adults ranges from 1% to 23%, which has been attributed to varying cutoff values used to define arterial stenosis and differences in diagnostic methods [1]. In pediatric patients, data on TRAS are scarce, with a reported prevalence of 5-9% [2,3]. A retrospective single-center study involving 216 children found a TRAS prevalence of 4.6% among those transplanted between 2001 and 2011, with hypertensive children after kidney transplantation (KTx) showing a higher prevalence, ranging from 5% to 15% [4].

Since hypertension is a common complication in pediatric patients - occurring in 71.7% and 61% of cases at five and 10 years post-KTx, respectively [5] - there is a clear need for careful evaluation of TRAS in this population. TRAS is the most frequent blood vessel-related complication following KTx, typically presenting between three months and two years after the procedure, although it can occur at any time post-surgery [6,7]. The timing and localization of renal artery stenosis may vary due to different underlying causes.

Well-documented risk factors for TRAS include surgical technique, prolonged ischemia time, delayed graft function (DGF), cytomegalovirus (CMV) infection, and humoral rejection [8]. The increased incidence of renal artery stenosis in deceased donor transplants compared to living-related transplants may be explained by cold ischemia prolongation and DGF causing endothelial damage and fibrosis [9]. CMV infection is also a recognized risk factor, although its exact molecular mechanism remains unclear. It is believed to be linked to the mitogenic effects of the viral cytomegalic gene product on vascular intima, promoting the proliferation and accumulation of smooth muscle cells [10]. Furthermore, post-anastomotic TRAS has been associated with the presence of de novo class II donor-specific antibodies [11].

This retrospective study aims to analyze the prevalence, clinical characteristics, risk factors, and outcomes of TRAS in children and adolescents who underwent KTx at our tertiary healthcare center.

## Materials and methods

Study design

This retrospective study was conducted at the Department of Nephrology and the Department of Interventional Cardiology at the University Children’s Hospital in Belgrade, Serbia. It was approved by the hospital’s Ethics Committee (decision number 16/23). Informed consent was obtained from a parent or legal guardian for each patient.

Subjects and eligibility

From June 2001 to February 2023, all patients who underwent KTx at our institution, the only pediatric KTx center in Serbia, were assessed for the presence of TRAS. This review included a total of 91 patients who underwent 95 KTx procedures. Patients experiencing allograft failure within the first three weeks after the KTx procedure were excluded from the study.

Three recipients who underwent two KTx procedures were included only after their second successful transplantation, as their initial grafts failed due to arterial or venous thrombosis. Additionally, two recipients were excluded because of arterial kinking or primary graft nonfunction; both received a second KTx abroad. Consequently, the study population consisted of 89 patients (39 females and 50 males) and 90 KTx procedures.

Suspicion of TRAS and the subsequent indication for angiography were based on the presence of de novo or refractory hypertension, defined as arterial blood pressure exceeding the 95th percentile despite the use of at least two antihypertensive medications. Other criteria included a decline in renal function without an identifiable cause and abnormalities detected during Doppler ultrasound (US) analysis. The US findings suggestive of TRAS included peak systolic velocities (PSV) greater than 200 cm/s, PSV increases of more than 50%, resistance indices exceeding 0.8, stenosis/pre-stenotic velocity gradients greater than 2:1, and parvus-tardus waveform with systolic acceleration lasting longer than 0.1 seconds [12].

Endovascular procedure

Endovascular procedures were performed using access through either the ipsilateral or contralateral common femoral artery. Angiography of the pelvic and transplanted renal artery was conducted using iodinated contrast (iopromide). Following this, selective catheterization of the donor renal artery anastomosis and angiography of the transplant renal artery in at least one oblique view were performed. A luminal diameter reduction of 50% or more during angiography was considered significant.

Patients with significant stenosis were treated with percutaneous transluminal angioplasty (PTA) or stent placement. During angiography, the TRAS diameter was measured for each patient. In cases where necessary, systemic heparinization was administered using unfractionated heparin (50 UI/kg), followed by lesion transposition using a 0.014 or 0.021 guidewire. The balloon catheters used during PTA procedures included Monorail NC Quantum Apex (Boston, MA, USA) with diameters of 5 × 15 mm, 6 × 20 mm, and 7 × 20 mm, as well as high-pressure Z med II catheters (4 × 20 mm). The maximum balloon inflation pressure applied was 16 atm. Treatment decisions, including catheter selection, were made at the discretion of the interventional physician. After the procedure, patients were monitored for one to two days in the transplant department.

Clinical variables and outcomes

The collected data included patient age, gender, primary renal disease, donor age, incidence of DGF, clinical signs, Doppler US and angiography findings, and the type of intervention performed. Clinical outcomes were assessed by recording the number of antihypertensive medications used, estimated glomerular filtration rate (eGFR), and blood pressure values measured immediately before PTA, two weeks after PTA, a minimum of three months post-PTA, and at the end of the follow-up period.

The blood pressure index was used to evaluate the severity of blood pressure elevation. It was calculated by dividing the average office-measured blood pressure by the 95th percentile of blood pressure specific to each patient [5]. DGF was defined as the need for hemodialysis within the first seven days following KTx [13]. CMV infection was diagnosed using a polymerase chain reaction, and all R-transplanted recipients who received grafts from D+ donors for CMV underwent a 12-week course of oral valganciclovir to prevent CMV infection.

The technical success of PTA was verified through Doppler US and was defined as a complete absence of or less than 30% residual stenosis. Clinical success was characterized by improved renal function, indicated by lower creatinine levels and enhanced eGFR in cases of renal dysfunction, or by better blood pressure control with reduced use of antihypertensive medications in patients with arterial hypertension caused by TRAS.

Statistical analysis

The normality of distribution for continuous variables was assessed using the Kolmogorov-Smirnov test with Lilliefors correction. Continuous data were reported as mean ± SD or median with IQR, while categorical variables were presented as frequencies and percentages. Comparisons between groups were conducted using parametric paired t-tests and nonparametric Wilcoxon rank-sum tests. Statistical analyses were performed using IBM SPSS Statistics for Windows, Version 27.0 (Released 2020; IBM Corp., Armonk, NY, USA).

## Results

From the cohort of transplanted patients at our institution, TRAS developed in seven out of 90 transplanted cases (7.8%): five out of 33 (15.2%) of cadaveric recipients and two out of 57 (3.5%) of living-related recipients (Table 1). The primary renal diseases among these seven cases included congenital anomalies of the kidneys and urinary tract (three cases), Alport syndrome (two cases), Wilms tumor, and Jeune syndrome (one case each).

**Table 1:** Demographics, transplant follow-up, and CMV status of patients with TRAS ACR, acute cellular rejection; Bx, biopsy; CAKUT, congenital anomaly of kidney and urinary tract; C, cadaveric; CMV, cytomegalovirus; CMV TIN, CMV-related tubulointerstitial nephritis; DGF, delayed graft function; KTx, kidney transplantation; L, living; PRN, primary renal disease; TRAS, transplant renal artery stenosis

Patient	Gender	Age (year)	PRN	Donor type/age (year)	Anastomosis	CMV mismatch	DGF	Bx	Time from KTx to Bx (months)	PCR CMV	CMV status and time from KTx
1	F	17.92	CAKUT	C/44	End-to-side	No	No	ACR	4.5	Negative	-
2	M	11.25	CAKUT	L/29	End-to-end	No	No	No	-	Negative	-
3	F	13.17	Wilms tumor	C/29	End-to-end	No	Yes	No	-	Negative	-
4	M	16.45	Alport syndrome	L/38	End-to-end	No	No	No rejection	1.3	Negative	-
5	M	18.13	Alport syndrome	C/64	End-to-end	Yes	No	CMV TIN	6	Positive	4.5 months
6	F	17.29	CAKUT	C/33	End-to-end	No	Yes	No	-	Positive	3 months
7	M	6.75	Jeune syndrome	C/41	End-to-end	Yes	No	No	-	Negative	-

Noninvasive imaging and clinical findings suggested TRAS in eight patients, all of whom underwent angiography using the Seldinger technique. One patient underwent only diagnostic angiography without intervention due to mild stenosis caused by anastomotic vessel angulation without flow disturbance; this patient was excluded from the final analysis. Ultimately, TRAS was angiographically confirmed in seven cases, all of which were treated by PTA or stent placement.

In six patients, systemic heparinization with unfractionated heparin (50 UI/kg) was administered following confirmation of stenosis via angiography. Two of the seven patients required additional re-intervention, resulting in a total of nine procedures. Two patients were treated with stent implantation: one was a girl with post-anastomotic diffuse narrowing of the donor artery immediately after failed PTA with a balloon catheter, and the other was a boy who underwent stent implantation as the primary treatment for multiple post-anastomotic stenoses.

For these stent placements, two stents were used for each patient: a 4 × 23 mm Bx Sonic (Cordis Corporation, Johnson & Johnson, Miami, FL, USA) and a 5 × 20 mm Liberte (Boston Scientific Corporation, Natick, MA, USA) for one case; and a 4.5 × 15 mm and 4.5 × 12 mm Rx Herculink Elite Renal Stent System (Abbott, Lake County, Illinois, USA) for the other. Both patients who underwent stent angioplasty received aspirin therapy (3-5 mg/kg) for a six-month period.

All arterial anastomoses were created on the iliac arteries. One case involved an end-to-side anastomosis on the external iliac artery, while the remaining cases involved end-to-end anastomoses on the internal iliac artery. Two recipients experienced vascular complications during KTx surgery, specifically perianastomotic bleeding requiring additional vessel suturing.

**Figure 1:**
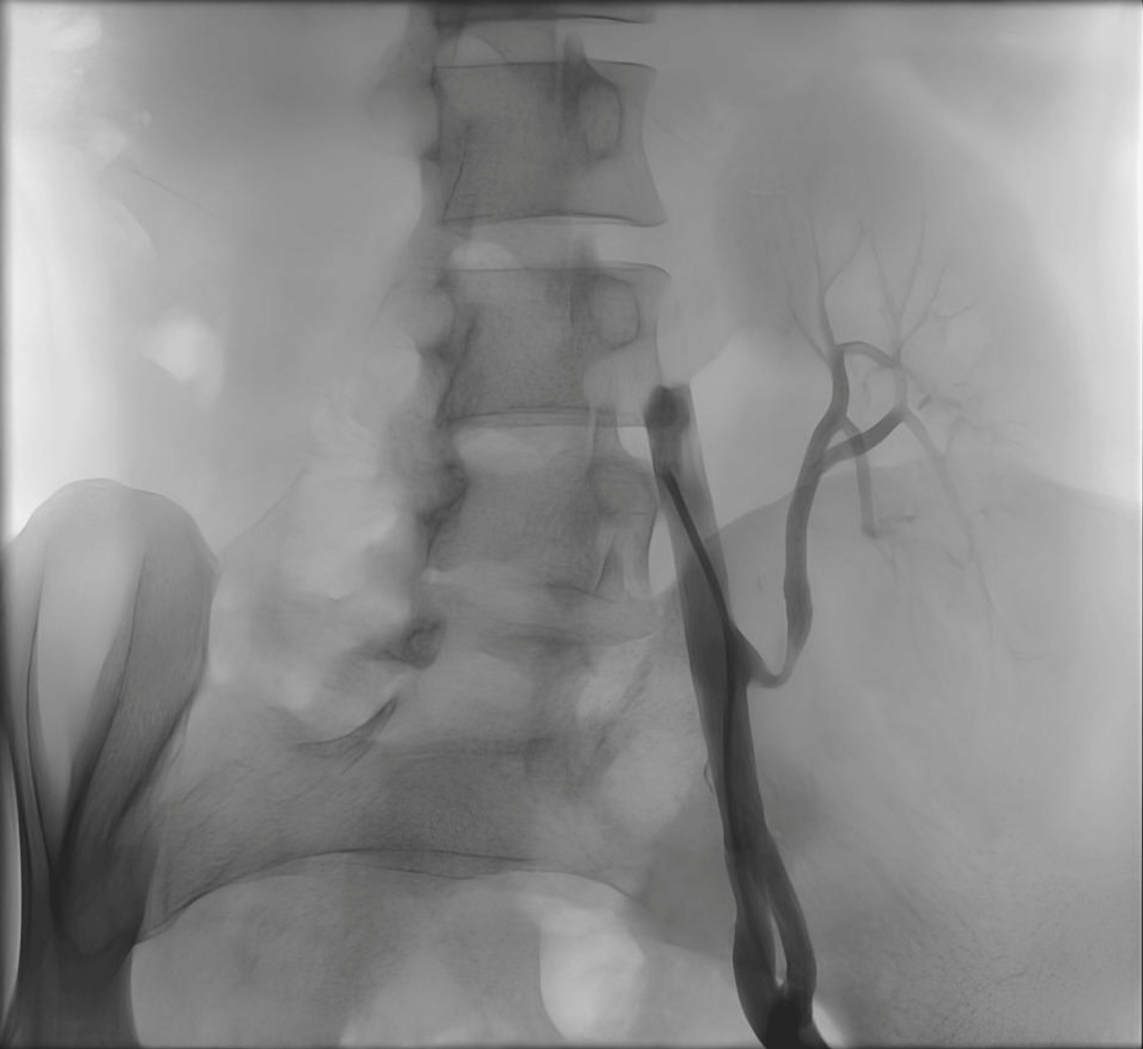
Patient No. 2: Visible stenosis angiography finding before the PTA with stent placement PTA, percutaneous transluminal angioplasty

**Figure 2:**
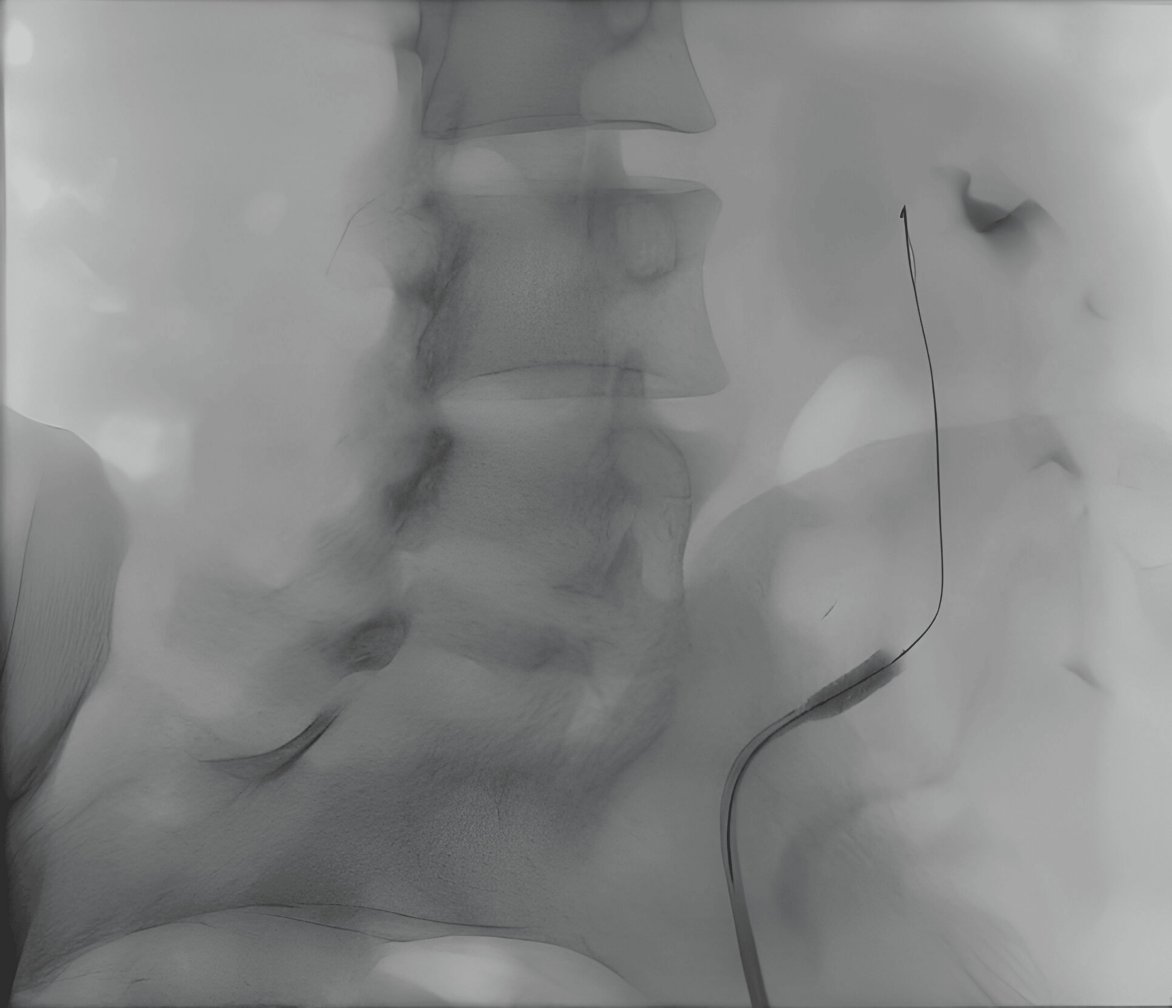
Patient No. 2: Angiogram during the first stent placement

**Figure 3:**
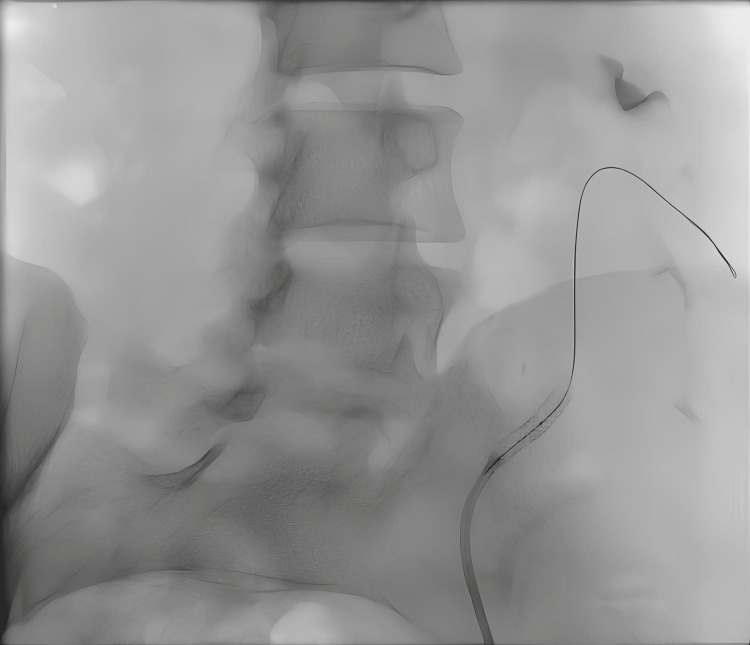
Patient No. 2: Angiogram during the second stent placement

**Figure 4:**
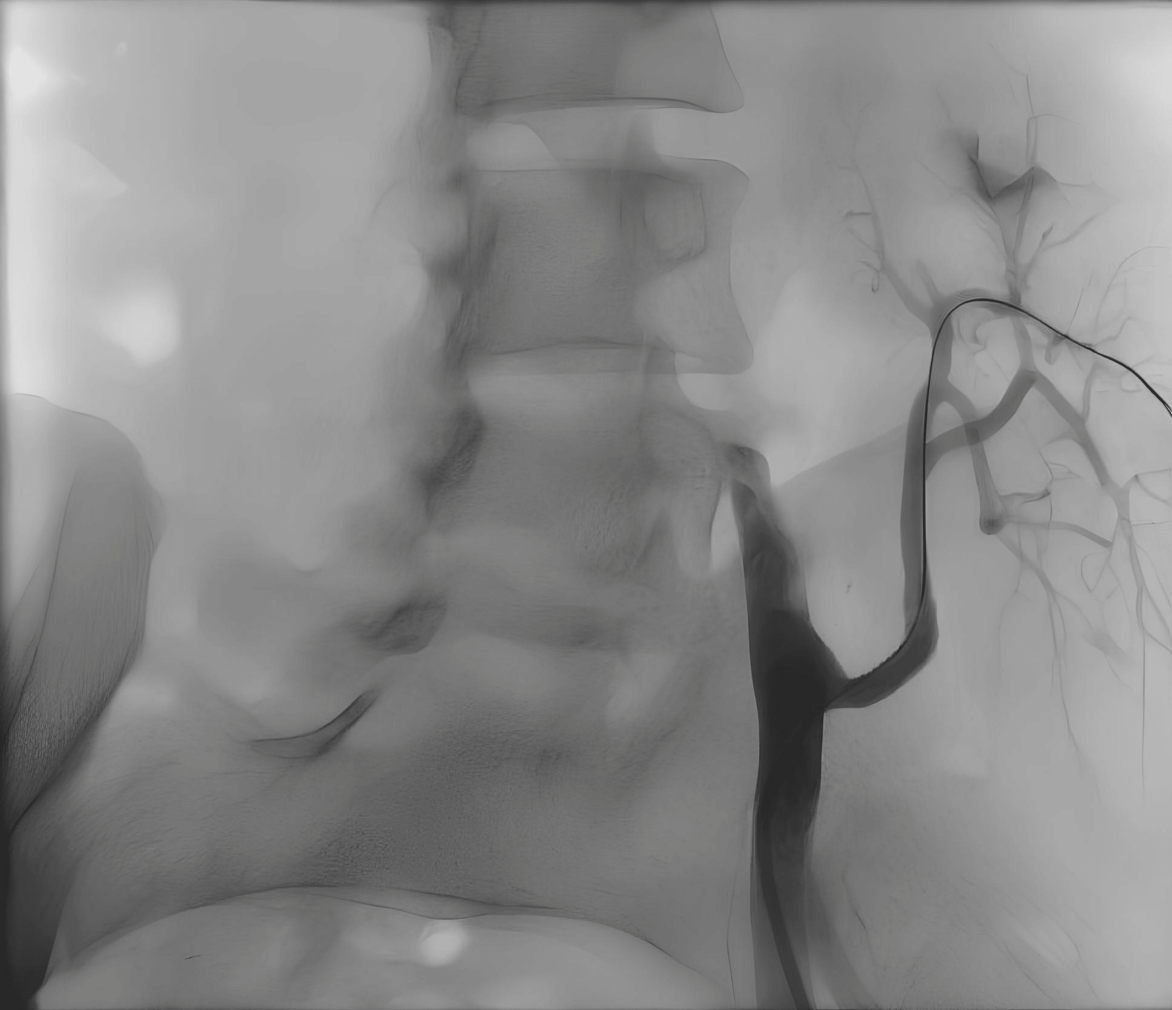
Patient No. 2: Angiography finding after the PTA with stent placements PTA, percutaneous transluminal angioplasty

Of the seven patients diagnosed with TRAS and treated with endovascular intervention, balloon angioplasty was performed in five cases, stent placement was the primary treatment in one case, and another case involved balloon angioplasty followed by two stent placements during the same procedure (Figure 1, Figure 2, Figure 3, Figure 4). The mean patient age was 14.42 ± 4.33 years, and the mean weight was 40.30 ± 19.20 kg. The median time to diagnosis following KTx was 3.5 months (IQR: 1.0-5.0).

The majority of patients with TRAS (71.4%) received grafts from deceased donors (five out of seven). Of the seven patients undergoing endovascular treatment, all but one presented with resistant systemic hypertension. The only clinical finding in the normotensive patient was an audible abdominal bruit, which was also detected in five out of seven patients upon clinical examination.

**Figure 5:**
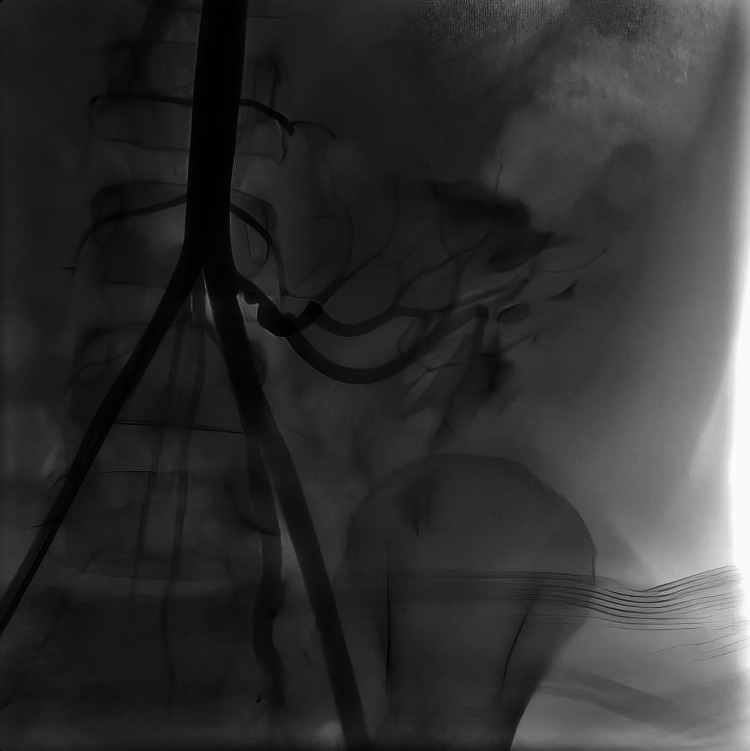
Patient No. 7: Visible stenosis angiography finding before the PTA PTA, percutaneous transluminal angioplasty

**Figure 6:**
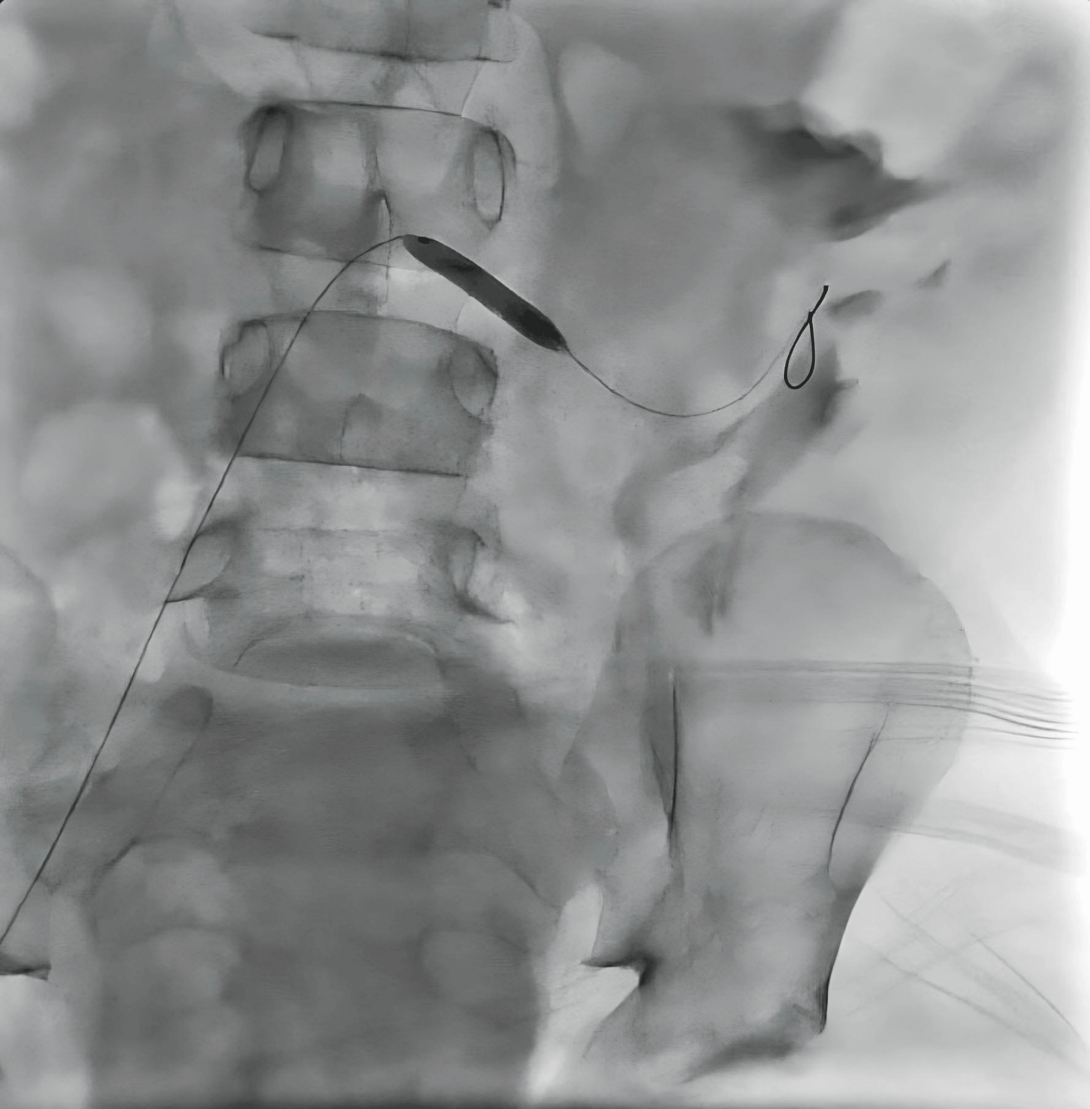
Patient No. 7: Angiogram during the balloon angioplasty

**Figure 7:**
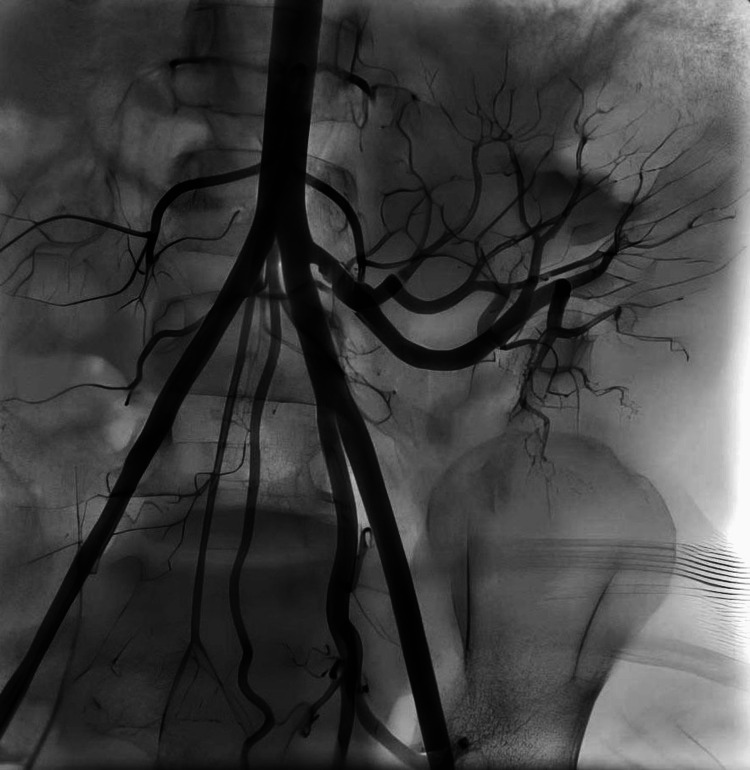
Patient No. 7: Angiography finding after the PTA PTA, percutaneous transluminal angioplasty

Regarding anastomotic development, three of the seven patients exhibited anastomotic stenoses, while two showed distal stenosis located 1.5-2.0 cm distal to the anastomosis (Figure 5, Figure 6, Figure 7). Additionally, one patient presented with double stenosis in the main artery, involving anastomotic and distal luminal narrowing estimated at 65% and 48%, respectively. Another patient had multiple distal stenoses.

Two patients tested positive for CMV via polymerase chain reaction, with viral loads of 470,000 and 2,887 copies/ml, respectively, within one month of arteriography. The patient with the higher viral load exhibited multiple segmental stenoses indicative of vasculitis, necessitating stent placement as the primary intervention without PTA attempts. Within a month of the procedure, this patient developed a complete clinical laboratory manifestation of hemophagocytic lymphohistiocytosis, possibly triggered by a CMV mismatch between the donor and recipient.

**Figure 8:**
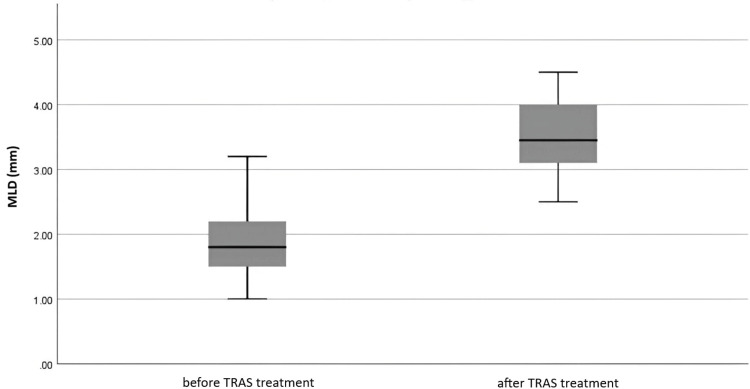
MLD values before and after TRAS treatment (n = 7) MLD, minimal lumen diameter; TRAS, transplant renal artery stenosis

Only one patient underwent an allograft biopsy within a month following arteriography, and the findings showed no signs of rejection (Table 2). Two of the seven patients experienced DGF. All patients who underwent intervention achieved technical success, demonstrated by a significant improvement in minimal lumen diameter (MLD) (Figure 8, Table 3).

**Table 2:** Clinical, angiographic, and treatment data of patients with TRAS CKD, chronic kidney disease; CMV HLH, cytomegalovirus-related hemophagocytic lymphohistiocytosis; Cr, creatinine; DSA, donor-specific antibodies; Dx, diagnosis; HTN, hypertension; KTx, kidney transplantation; PTA, percutaneous transluminal angioplasty; PTDM, posttransplant diabetes mellitus; Rx, treatment; TRAS, transplant renal artery stenosis; US, ultrasound

Patient	Clinical signs	Doppler US	Angiography	Luminal narrowing	Time from KTx to Dx (months)	Rx	Clinical course	Restenosis (months)	Total clinical follow-up (months)	Outcome
1	↑HTN, seizures, abdominal bruit	Positive	1.5 cm after anastomosis	68.90%	11	Stent	-	No	21	Good, well-controlled HTN
2	↑HTN, seizures, abdominal bruit	Positive	Anastomotic stenosis	75.96%	1	PTA	De novo DSA class II	Yes, at 15	44	Good
3	Abdominal bruit	Positive	Anastomotic stenosis	79.16%	2.5	PTA	-	No	38	Good
4	↑Cr, ↑HTN	Negative	Anastomotic stenosis	68.96%	1.5	PTA	De novo DSA class II	Yes, at 4	104	Good
5	↑Cr, ↑HTN, abdominal bruit	Positive	Anastomosis, multiple stenoses	51.11%	0.5	Stent	CMV HLH	No	12	CKD
6	↑HTN, abdominal bruit	Positive	Anastomotic + distal stenosis	65.08%, 47.61%	3.5	PTA	PTDM	No	6	Good
7	↑HTN	Positive	After anastomosis	50.00%	5	PTA	-	No	60	-

**Table 3:** Technical and clinical outcome of PTA and stenting p*, significance between different measurement time points and baseline measurements for total interventions **, missing values AHT N, antihypertensive therapy drug number; DBPi, diastolic blood pressure index; eGFR, estimated glomerular filtration rate; MAP, mean arterial pressure; MLD, mean luminal diameter; PTA, percutaneous transluminal angioplasty; SBPi, systolic blood pressure index

Measurement	Balloon angioplasty (n = 5) mean ± SD or med (IQR)	Stenting (n = 2) mean ± SD or m(IQR)	Total (n = 7) mean ± SD or med (IQR)	p*
MLD (mm)				
Baseline	1.8 (1.3-2.6)	1.9 (1.5-1.9)	1.8 (1.4-2.4)	>0.99
Post-treatment	3.3 (3.1-4.0)	4.5 (4.5-4.5)	3.5 (3.1-4.3)	0.01
MAP (mmHg)				
Baseline	110.04 ± 6.38	126.67 ± 6.90	113.74 ± 9.71	>0.99
Two weeks post-treatment	84.55 ± 14.94	99.48 ± 8.74	88.28 ± 14.77	<0.01
Three months post-treatment	95.00 ± 17.32	87.50 ± 15.32	93.33 ± 16.29	0.66
One year post-treatment	80.83 ± 10.95	91.67 ± 2.35	83.54 ± 10.56	0.04
Two years post-treatment	80.66 ± 17.27	**	80.66 ± 17.27	0.13
End of follow-up	84.15 ± 10.21	81.67 ± 0.00	83.80 ± 9.37	0.11
SBPi				
Baseline	1.26 ± 0.13	1.34 ± 0.03	1.28 ± 0.12	>0.99
Two weeks post-treatment	0.9 (0.9-1.0)	1.1 (1.0-1.1)	0.9 (0.8-1.1)	0.02
Three months post-treatment	1.05 ± 0.16	1.02 ± 0.13	1.04 ± 0.15	<0.01
One year post-treatment	0.91 ± 0.14	1.00 ± 0.06	0.93 ± 0.13	<0.01
Two years post-treatment	0.89 ± 0.15	**	0.89 ± 0.15	<0.01
End of follow-up	0.94 ± 0.11	**	0.92 ± 0.11	<0.01
DBPi				
Baseline	1.19 ± 0.12	1.46 ± 0.04	1.25 ± 0.15	>0.99
Two weeks post-treatment	0.95 ± 0.17	1.13 ± 0.14	0.99 ± 0.17	0.02
Three months post-treatment	1.06 ± 0.17	0.95 ± 0.28	1.04 ± 0.18	0.03
One year post-treatment	0.87 ± 0.11	1.01 ± 0.01	0.91 ± 0.12	0.02
Two years post-treatment	0.89 ± 0.28	**	0.89 ± 0.28	0.01
End of follow-up	0.91 ± 0.12	0.88 ± 0.00	0.91 ± 0.11	0.02
eGFR (mL/min/1.73m²)				
Baseline	74.19 ± 14.67	62.78 ± 6.90	71.66 ± 13.88	>0.99
Two weeks post-treatment	76.96 ± 14.96	71.40 ± 16.51	75.72 ± 14.42	0.57
Three months post-treatment	79.78 ± 15.16	74.27 ± 10.20	78.56 ± 13.83	0.05
One year post-treatment	88.18 ± 34.09	84.47 ± 22.88	87.26 ± 30.13	0.14
Two years post-treatment	96.09 ± 14.84	**	96.09 ± 14.84	0.23
End of follow-up	73.18 ± 29.12	64.85 ± 0.00	71.99 ± 26.77	0.23
AHT N (antihypertensive medications)				
Baseline	2.57 ± 1.27	3.00 ± 0.00	2.67 ± 1.12	>0.99
Two weeks post-treatment	1.14 ± 0.90	0.50 ± 0.71	1.00 ± 0.87	0.01
Three months post-treatment	2.00 (0.00-2.00)	1.50 (1.00-1.50)	2.00 (0.50-2.00)	0.01
One year post-treatment	1.50 ± 1.38	2.00 ± 1.40	1.63 ± 1.30	0.01
Two years post-treatment	1.20 ± 1.30	**	1.20 ± 1.30	0.02
End of follow-up	1.83 ± 1.33	2.00 ± 0.00	1.86 ± 1.22	0.05

Both balloon angioplasty and stent placement yielded considerable MLD improvement, with all interventions resulting in an MLD increase of over 53.8% post-procedure. The mean stenosis rates before and after treatment were 68.03% and 28.37%, respectively. Clinical success was achieved in all cases, as reflected by slightly improved eGFR values (Table 3), although the improvement was not statistically significant.

**Figure 9:**
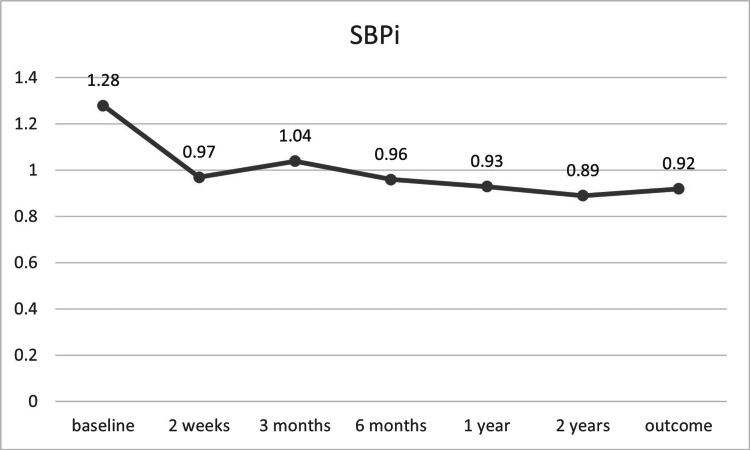
SBPi before and after TRAS treatment (n = 7) SBPi, systolic blood pressure index; TRAS, transplant renal artery stenosis

**Figure 10:**
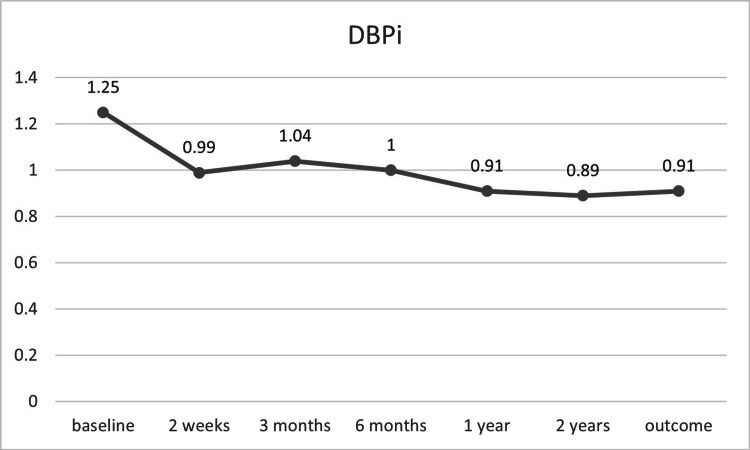
DBPi before and after TRAS treatment (n = 7) DBPi, diastolic blood pressure index; TRAS, transplant renal artery stenosis

A significant decrease in average mean arterial pressure was observed one year after intervention (p = 0.04; Table 3). Additionally, sustained significant reductions in systolic and diastolic blood pressure indices were noted following the interventions (Figure 9, Figure 10).

The median number of antihypertensive medications used was reduced from 2.67 ± 1.12 pre-intervention to 1.00 ± 0.87 two weeks post-intervention (p = 0.011). This improvement in antihypertensive drug usage was maintained after one year, two years, and at the most recent follow-up (p = 0.01, 0.02, and 0.05, respectively).

During the mean follow-up period of 56.86 ± 45.76 months, two patients experienced re-stenosis and were referred to the catheterization laboratory due to worsening blood pressure levels. These patients were initially part of the balloon angioplasty group and underwent repeated intervention. One patient underwent a second balloon angioplasty 15 months after his first procedure, while the other patient after five months. The overall restenosis rate was 28.6% (two out of seven patients). Among those who underwent balloon angioplasty, the restenosis rate was 40% (two out of five patients). No restenosis was observed after stent angioplasty. In both patients, re-stenosis responded favorably to balloon angioplasty, without a stent placement requirement. The post-intervention course of two patients was complicated by chronic humoral rejection (with de novo donor-specific antibodies development) of the renal allograft after 1.5 years and six years, respectively. During these nine interventions, a small perianastomotic hematoma formed in only one patient after the first angioplasty, and the patient subsequently did well with no further complications and with hematoma disappearance over time. There were no other intra-procedural complications observed.

At the latest follow-up with available data for our analysis (56.86 ± 45.76 months), all patients had functioning grafts and experienced no complications related to PTA ± stenting.

## Discussion

TRAS is increasingly identified due to the widespread use of noninvasive screening techniques such as Doppler US and magnetic resonance angiography [14]. Despite improvements in preoperative evaluations, advancements in KTx techniques, and reduced surgical complications, vascular issues remain the most common complications following KTx procedures, with reported TRAS prevalence ranging from 1% to 23% among all KTx recipients [1]. This wide range in reported prevalence largely results from variations in diagnostic methods and differing definitions of the arterial stenosis cutoff value. For example, Wong et al. [15] reported a TRAS prevalence of 2.4% before and 12.4% after implementing “screening” color-Doppler US. In our study, the prevalence of TRAS was 7.8%, comparable to or higher than rates reported in other studies [1,4,16-18]. The relatively higher prevalence in our cohort may be attributed to the larger proportion of patients included after introducing the screening program in 2011, unlike many other studies conducted before this period. Additionally, our threshold for defining hemodynamically significant stenosis (≥50%) is lower than what most studies use. The criteria for defining hemodynamically significant TRAS remain unstandardized, with researchers employing arterial lumen narrowing thresholds ranging from over 50% to over 80% [8].

In the absence of rejection, ureteric obstruction, or infection, vascular complications often present as worsening or refractory hypertension, with or without graft dysfunction. The prevalence of renal artery stenosis in children with elevated blood pressure following KTx ranges from 5% to 15% [19]. Since hypertension is a common complication in pediatric KTx patients, affecting 58-89% of cases [5,20], it is crucial to consider vascular complications as a potential cause of hypertension. Our analysis revealed that new-onset or worsening hypertension, associated with Doppler US abnormalities (PSV >200 cm/s), was a primary clinical manifestation, observed in more than 85% of cases (Table 2).

These findings are consistent with those reported by Ren et al., who noted refractory hypertension in 81.8% of TRAS patients, although their cohort had a higher incidence of graft dysfunction (72.7%) [21]. In our study, only two out of seven TRAS patients (28.6%) had elevated serum creatinine, which aligns with findings by Li et al., who reported this in four out of 14 patients with TRAS [22]. This observation differs from other studies that identified renal dysfunction as the most common clinical indicator of TRAS [4,18,23]. The discrepancy may be due to serum creatinine being a less reliable indicator of kidney function in younger patients.

Nonetheless, TRAS can also be clinically silent or present with an abdominal bruit, as noted in one of our patients. Therefore, regular screening of KTx patients using Doppler US imaging - conducted monthly for the first six months and then twice a year - should be a fundamental part of the follow-up protocol.

The location of vessel narrowing and the timing of disease onset can provide clues about the underlying causes. For example, anastomotic stenosis is most commonly associated with surgical techniques used during organ harvesting and transplantation, typically occurring early after the transplant. Suture-related complications are more likely in end-to-end anastomoses due to structural and diameter differences between donor and recipient vessels [24].

In our patient group, 57% of TRAS patients (three out of seven) had stenosis located at the anastomosis site, which aligns with findings from other studies [16,18,23]. Additionally, end-to-end anastomoses were predominant among our TRAS patients (six out of seven), which is consistent with findings reported by Ayvazoglu Soy et al. [25]. TRAS typically presents between three months and two years posttransplantation [6,7]. The mean onset of TRAS in our study was 107 days, comparable to the study by Valle et al. [18], but different from other reports [26,27].

Some researchers have noted a positive correlation between TRAS and cadaveric transplants [28]. The extended cold ischemia time required for cadaveric transplants may contribute to renal artery endothelial damage. Two decades ago, TRAS prevalence in cadaveric transplants was reported as 4.5% [29] and 6.5% [9] in separate studies. Conversely, a retrospective study of 1,000 living-related transplants from the same period showed a prevalence of 1.7% [30].

Patel et al. reported an overall TRAS prevalence of 3.1%, with higher rates in cadaveric transplants (4.1%) compared to living-related transplants (0.8%) [8]. Similarly, our study found a higher prevalence in cadaveric transplants (15.2%) compared to living-related transplants (3.5%). Stenoses that occur later, sometimes several years post-KTx, are often indicative of atherosclerotic disease or may be associated with rejection and/or CMV infection [4,9,16,23].

Pouria et al. identified a link between TRAS and CMV infection-induced large vessel damage in a study involving 917 KTx procedures [10]. Patel et al. also reported that two out of 26 patients with TRAS had positive CMV serology test results, with arteriography revealing multiple segmental stenoses suggestive of vasculitis [8]. These patients were successfully treated with anti-CMV medications.

In our study, two out of seven TRAS patients developed CMV antigenemia either immediately or 1.5 months after discontinuing CMV prophylaxis. Despite re-induction with valganciclovir, both patients required endovascular treatment-PTA and PTA with stent placement in a patient with multiple stenoses.

Angiography remains the gold standard for diagnosing TRAS. While clinical criteria for revascularization in cases of significant renal artery stenosis in native kidneys are well-established [31], clear guidelines for KTx recipients are still lacking. Generally, angiography-detected arterial diameter stenosis of ≤50% is not considered hemodynamically significant. Treatment of TRAS is essential since graft dysfunction and/or high blood pressure can compromise both renal graft and patient survival [32-34].

PTA, alone or combined with stenting, is widely accepted as the initial treatment for TRAS. Surgical intervention is reserved as a last resort when angioplasty proves unsuccessful [7]. The technical success rate of PTA exceeds 80%, accompanied by clinical success measured by improvements in hypertension and allograft function [35-37]. Patel et al. defined clinical success as a 15% reduction in serum creatinine and diastolic blood pressure without increasing the number of antihypertensive medications, or a 10% reduction in diastolic blood pressure with a decreased need for antihypertensive medications [8].

In our case series, we observed more than a 10% reduction in mean arterial pressure, with significant improvements in systolic and diastolic blood pressure throughout the follow-up period (Figure 9, Figure 10). A notable reduction in the number of antihypertensive drugs was also observed, unlike some previous reports (Table 3) [16,27]. Additionally, graft function showed improvement, with mean creatinine clearance increasing by more than 34% over two years, although statistical significance was not achieved (Table 3).

Among the six TRAS patients treated with PTA, two achieved optimal (>75%) luminal gain, and three achieved good (>50%) luminal gain, resulting in an overall success rate of 83.3%, consistent with other published studies [38,39]. In one case where the procedure was considered suboptimal, stent placement was performed during the same procedure. This patient and another initially treated with stent placement achieved optimal technical success after six and 12 months of follow-up, respectively.

At a mean follow-up of 56.9 months, two patients treated with PTA required reintervention due to restenosis detected by Doppler US at four and fifteen months post-primary PTA. Both patients also developed de novo donor-specific antibodies (class II), which may be a pathogenic risk factor for TRAS [11]. The success rate for re-intervention was 100%, with one patient achieving optimal (>75%) luminal gain and another achieving good (>50%) luminal gain.

The restenosis rate in our PTA group was 40%, which is similar to or slightly higher than those reported in other studies [8,40,41]. Restenosis rates for balloon angioplasty generally range from 21% to 35% in studies involving native kidneys [41,42]. Our overall restenosis rate was 28.6%, which aligns with recent publications [41,43,44]. A restenosis rate of 37% has been reported following stenting procedures in children with renovascular hypertension [44]. Notably, the two patients treated with stent angioplasty in our study experienced no restenosis during the follow-up period.

According to the literature, early complications such as bleeding, pseudoaneurysm formation, thrombosis, arterial dissection, hematoma, and intimal flap occur in approximately 10% of cases [16,45]. In our experience, one case developed a small perianastomotic hematoma following the first angioplasty, which resolved over time without further complications. This benign early complication rate of 11.1% (one out of nine endovascular procedures) aligns well with previous reports [16,44].

No late adverse events were detected, and six out of seven patients demonstrated good and stable graft function with normal Doppler US findings at the most recent follow-up. However, one patient exhibited a doubling of serum creatinine one year after the most recent follow-up due to severe CMV disease complicated by hemophagocytic lymphohistiocytosis syndrome.

This single-center analysis highlights a significant TRAS prevalence in pediatric KTx recipients, emphasizing the importance of routine screening for TRAS in all patients post-KTx, beginning with renal echo-color Doppler imaging. Close monitoring with Doppler US is particularly crucial for patients with cadaveric grafts and hypertension.

Our findings confirm that PTA and stenting are effective and safe therapeutic procedures for pediatric recipients, providing excellent medium-term outcomes regarding graft function and blood pressure control.

However, this study’s primary limitations are the small sample size and the lack of data on procedural radiation dose, aspects that should be carefully addressed in future research.

## Conclusions

Despite significant advancements in KTx, vascular complications remain a major cause of morbidity and mortality. Preventing technical issues at every stage of the transplant process and implementing careful, noninvasive post-operative monitoring are essential for early diagnosis and effective management. In our small cohort, the overall rate of TRAS was comparable to or higher than previously reported rates, with a higher prevalence in cadaveric transplants (15.2%) compared to living-related transplants (3.5%). The intermediate-term outcomes of PTA for treating TRAS were favorable, demonstrating safety and effectiveness consistent with results reported in the literature. However, the role of stent placement, particularly in growing children, and its impact on long-term patency require further investigation.
